# Supplementary material for: Bis-thiobarbiturates as Promising Xanthine Oxidase Inhibitors: Synthesis and Biological Evaluation
Source: Biomedicines. 2021 Oct 11;9(10):1443. doi: 10.3390/biomedicines9101443 (PMC8533253; doi:10.3390/biomedicines9101443)
Supplement: Supplementary file 1 [file biomedicines-09-01443-s001.zip › biomedicines-1400551-supplementary.pdf]

Supplementary Information

Bis-thiobarbiturates as Promising Xanthine Oxidase Inhibitors: Synthesis and Biological Evaluation

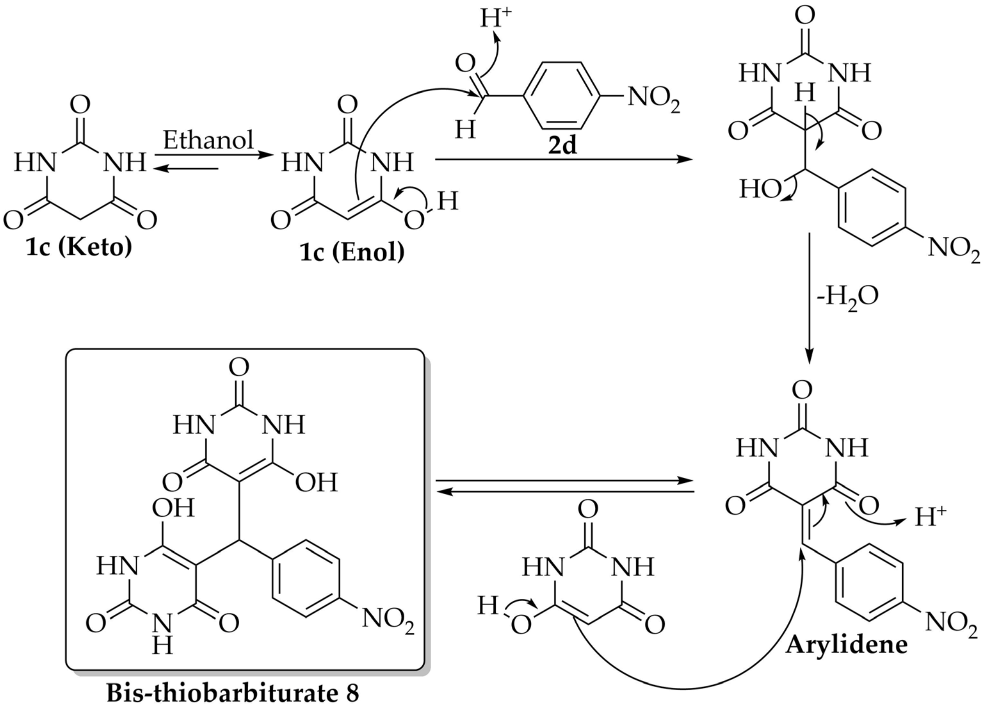

Scheme S1 – Chemical mechanism for the formation of bis-thiobarbiturate **8**.

Table S1 – Full *in vitro* data for XO inhibitory activity, DPPH radical scavenging strength and cytotoxicity effects on NHDF, Caco-2 and MCF-7 cells by bis-thiobarbiturates **3**-**7** and **9**-**19** and references Febuxostat, Allo, Trolox and 5-FU.[a]

Compound	XO inhibition (%)		DPPH (% scavenging at 30 μM)	Cytotoxicity (% cell viability at 30 μM)		
	30 μM	5 μM		NHDF	Caco-2	MCF-7
**3**	80.15 ± 0.68	10.42 ± 1.01	34.58 ± 0.96	88.65 ± 1.40	91.23 ± 9.87	89.96 ± 5.33
**4**	36.47 ± 2.95	-	42.64 ± 0.81	95.57 ± 7.26	98.58 ± 5.70	90.64 ± 3.92
**5**	86.12 ± 2.41	52.92 ± 4.02	34.76 ± 1.42	86.48 ± 5.92	62.52 ± 11.94	90.68 ± 4.06
**6**	92.25 ± 0.09	56.82 ± 2.16	34.99 ± 1.32	99.78 ± 5.43	100.13 ± 4.45	91.68 ± 1.48
**7**	20.86 ± 2.47	-	32.41 ± 0.26	96.60 ± 4.32	97.09 ± 3.63	98.66 ± 4.83
**9**	45.61 ± 1.69	-	39.63 ± 2.26	81.35 ± 8.87	67.49 ± 9.4	90.18 ± 5.70
**10**	23.53 ± 0.17	-	38.12 ± 1.56	57.11 ± 8.12	62.6 ± 7.74	100.16 ± 13.01
**11**	95.72 ± 1.49	80.92 ± 0.59	57.25 ± 0.98	94.27 ± 7.84	72.31 ± 9.76	92.15 ± 3.68
**12**	72.25 ± 5.65	-	42.92 ± 0.61	85.64 ± 7.17	90.88 ± 3.84	91.70 ± 4.91
**13**	89.73 ± 1.41	37.42 ± 2.55	29.90 ± 0.50	75.80 ± 2.41	95.43 ± 4.17	98.22 ± 3.25
**14**	90.70 ± 0.71	56.29 ± 1.21	28.04 ± 0.25	80.13 ± 7.69	97.2 ± 2.97	88.06 ± 3.25
**15**	14.92 ± 1.68	-	32.33 ± 0.48	91.18 ± 7.77	96.91 ± 4.73	91.25 ± 4.26
**16**	61.44 ± 1.22	-	31.35 ± 0.91	60.68 ± 1.72	95.36 ± 3.30	91.11 ± 2.00
**17**	90.75 ± 2.87	55.2 ± 1.63	40.90 ± 0.65	68.89 ± 9.33	88.72 ± 7.85	86.62 ± 5.87
**18**	98.85 ± 0.82	63.43 ± 2.1	31.04 ± 0.66	86.55 ± 2.38	94.68 ± 7.19	88.29 ± 3.29
**19**	91.68 ± 1.87	67.3 ± 1.27	3.83 ± 2.44	81.01 ± 3.57	80.71 ± 12.98	93.67 ± 5.68
**Febuxostat**	95.03 ± 0.91	93.88 ± 2.84	-	-	-	-
**Allo**	88.51 ± 3.64	26.93 ± 0.82	0.48 ± 0.21	-	-	-
**Trolox**	-	-	54.86 ± 0.98	-	-	-
**5-FU**	-	-	-	20.93 ± 0.83	37.06 ± 1.51	45.88 ± 4.72

[a] Results are expressed as average values ± standard deviation (SD) of at least two independent determinations.

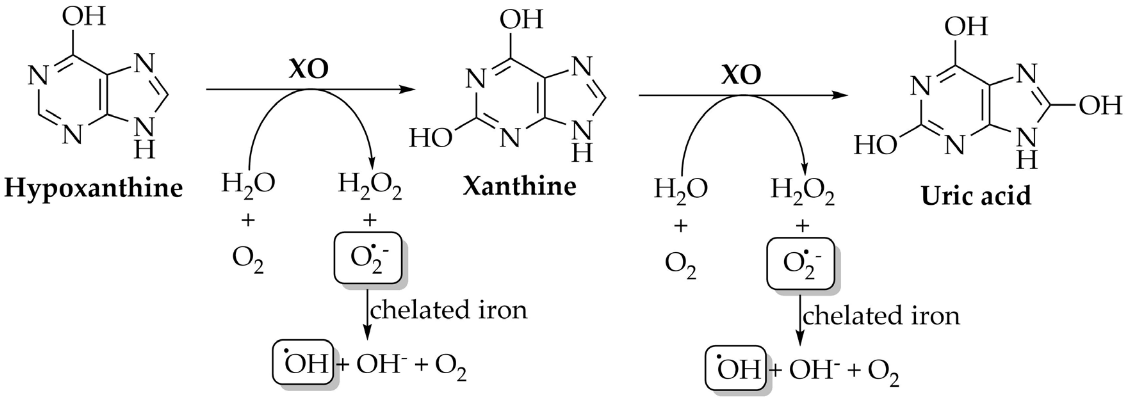

Scheme S2 – Diagram of XO oxidative hydroxylation of hypoxanthine over xanthine and uric acid, with reactive oxygen species (superoxide anion radical and hydroxyl radical) generation (adapted from Šmelcerović *et al*. [1[1]]).

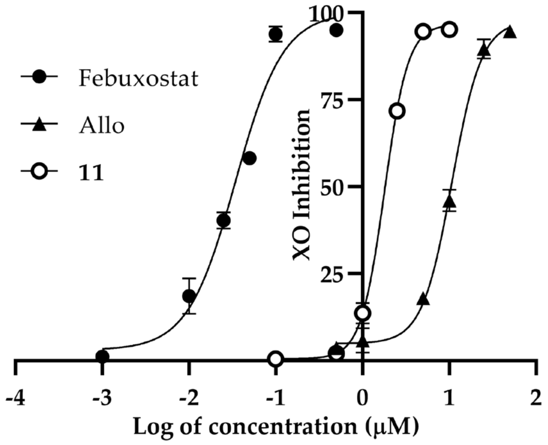

Figure S1 – IC50 curves for XO inhibition by Febuxostat, Allo and bis-thiobarbiturate **11**.

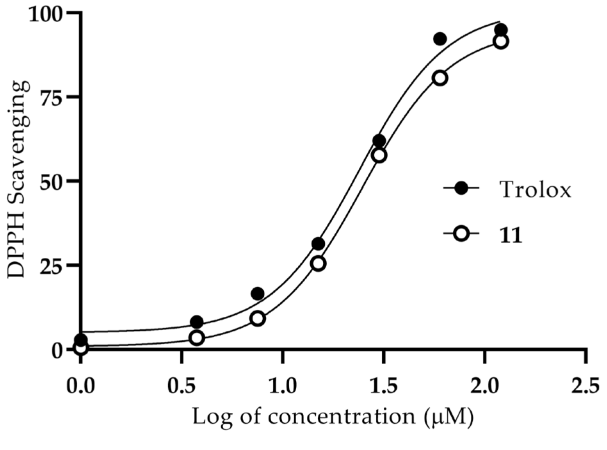

Figure S2 – IC50 curves for DPPH scavenging activity of Trolox and bis-thiobarbiturate **11**.

[1] Šmelcerović, A.; Tomović, K.; Šmelcerović, Ž.; Petronijević, Ž.; Kocić, G.; Tomašič, T.; Jakopin, Ž.; Anderluh, M. Xanthine oxidase inhibitors beyond allopurinol and febuxostat; an overview and selection of potential leads based on in silico calculated physico-chemical properties, predicted pharmacokinetics and toxicity. *Eur J Med Chem* **2017**, *135*, 491-516.

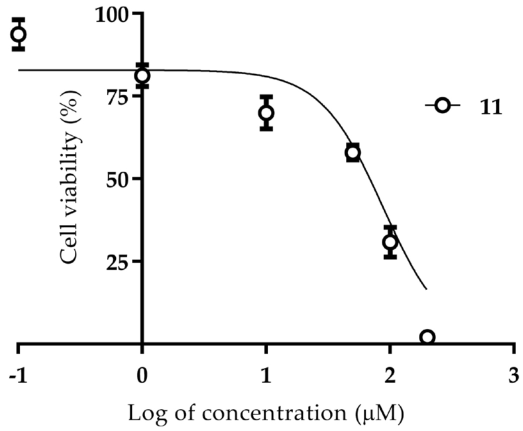

Figure S3 – IC_{50} curve for cytotoxicity of bis-thiobarbiturate **11** on NHDF cell line.

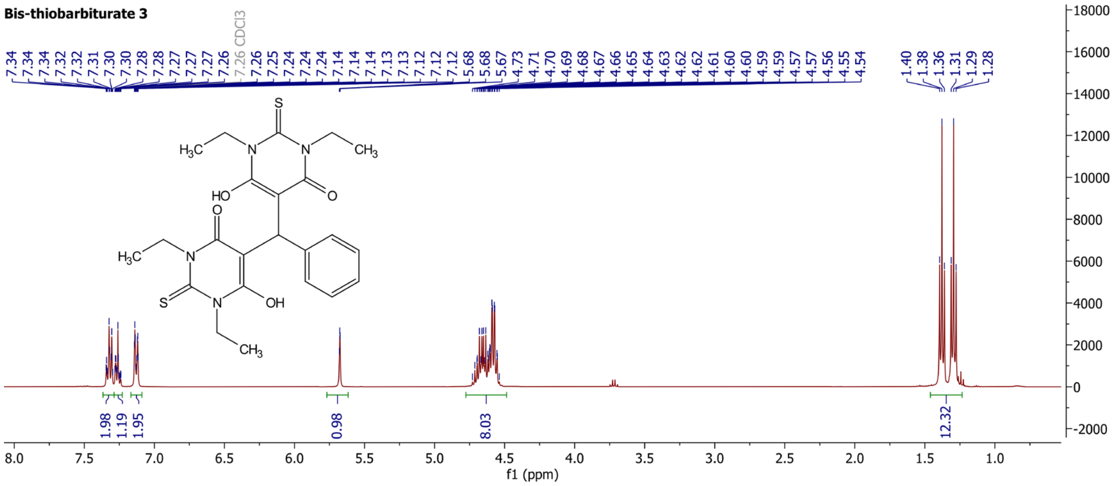

Figure S4 – ^{1}H NMR of bis-thiobarbiturate **3** in $CDCl_3$.

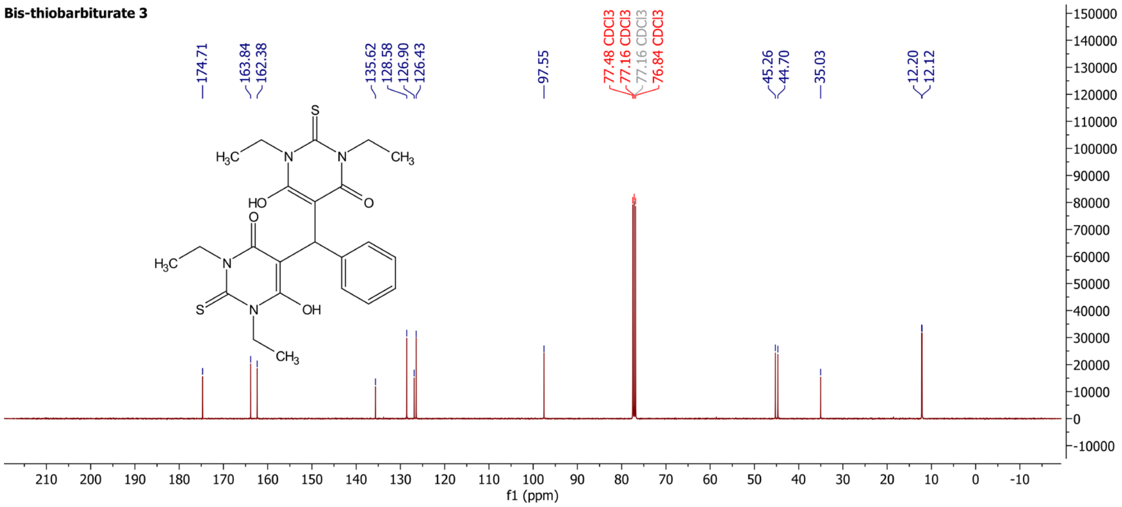

Figure S5 – ^{13}C NMR of bis-thiobarbiturate **3** in $CDCl_3$.

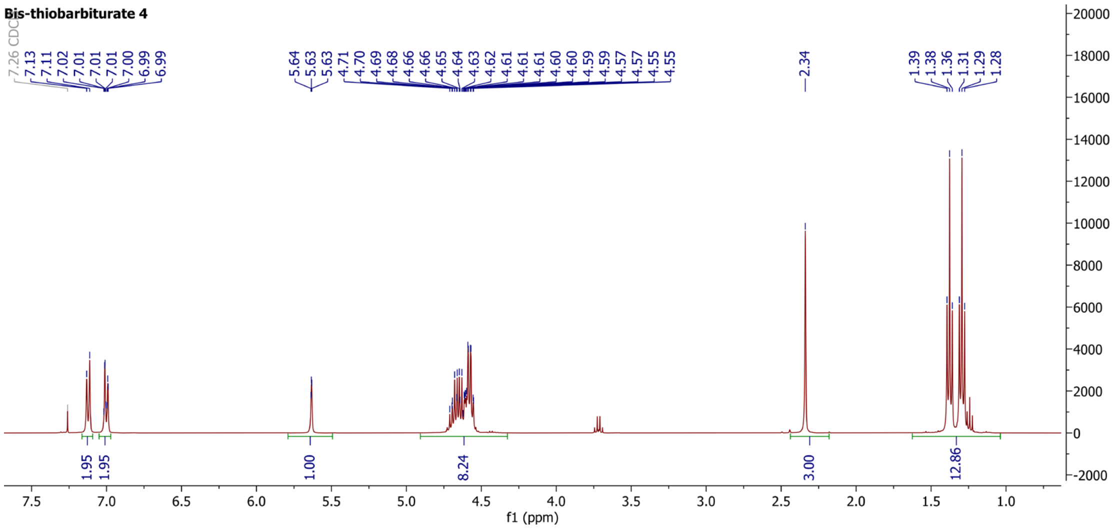

Figure S6 – ^{1}H NMR of bis-thiobarbiturate **4** in $CDCl_3$.

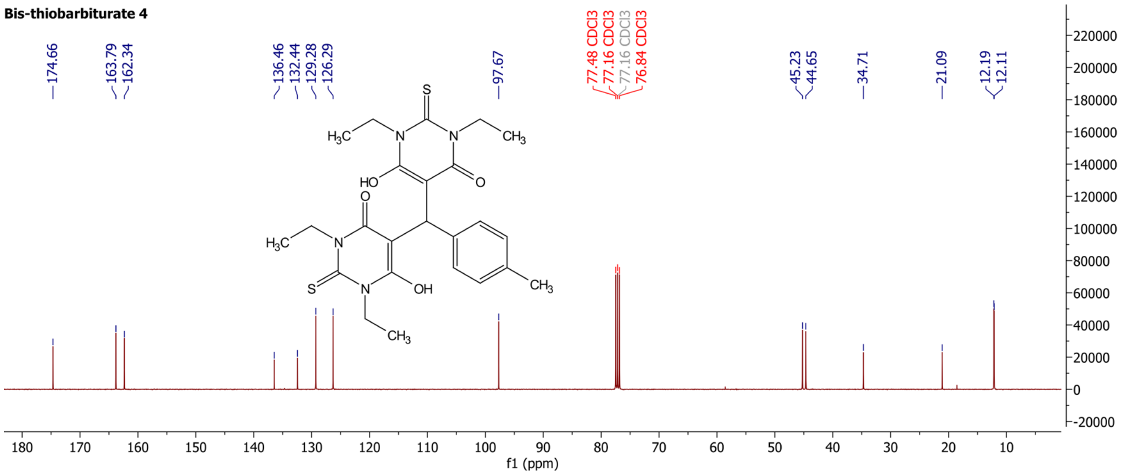

Figure S7 – ^{13}C NMR of bis-thiobarbiturate **4** in $CDCl_3$.

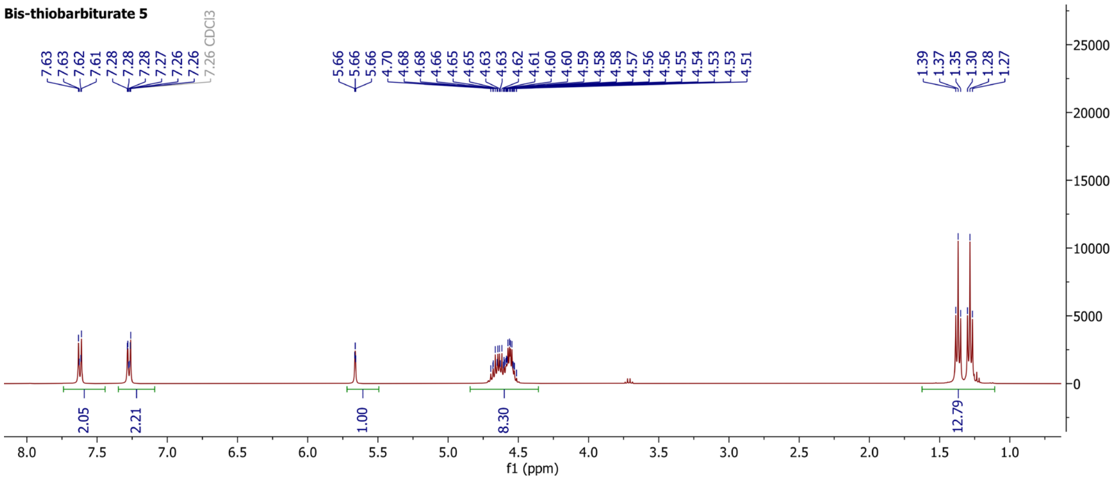

Figure S8 – ^{1}H NMR of bis-thiobarbiturate **5** in $CDCl_3$.

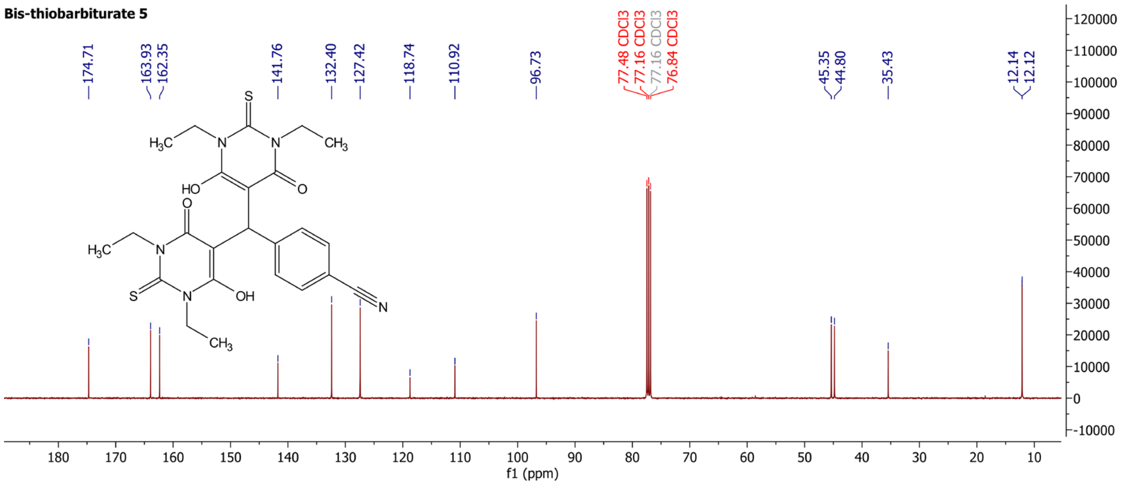

Figure S9 – ^{13}C NMR of bis-thiobarbiturate **5** in $CDCl_3$.

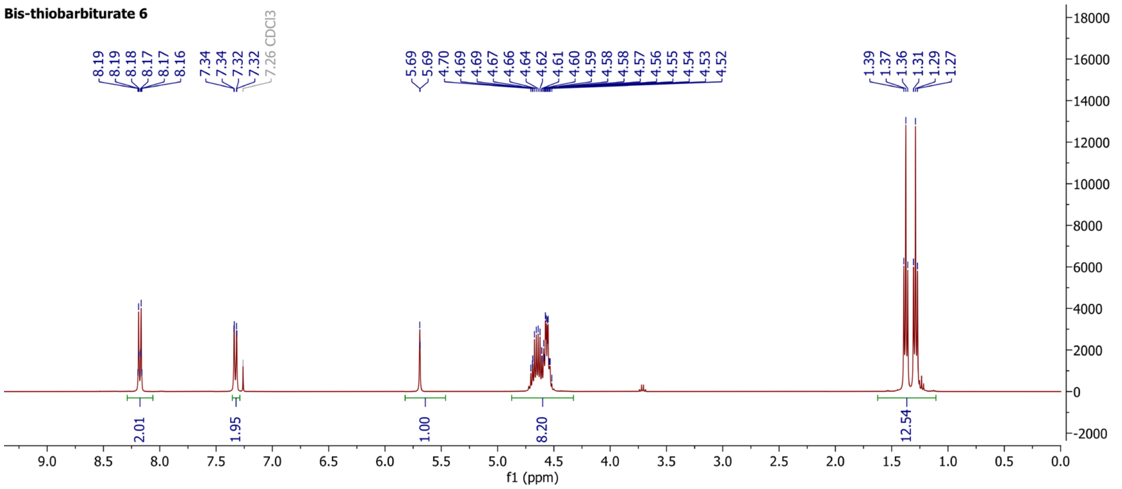

Figure S10 – ^{1}H NMR of bis-thiobarbiturate **6** in $CDCl_3$.

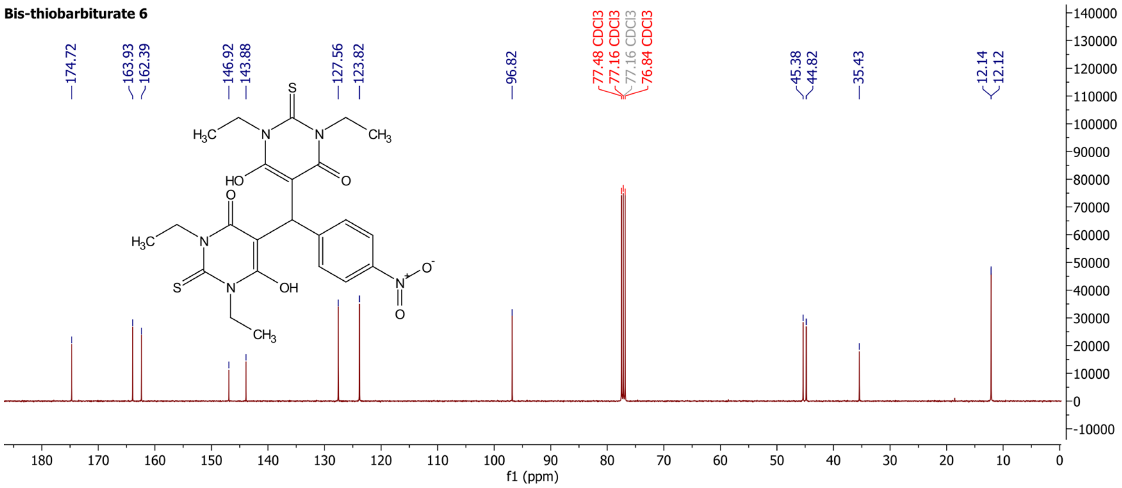

Figure S11 – ^{13}C NMR of bis-thiobarbiturate **6** in $CDCl_3$.

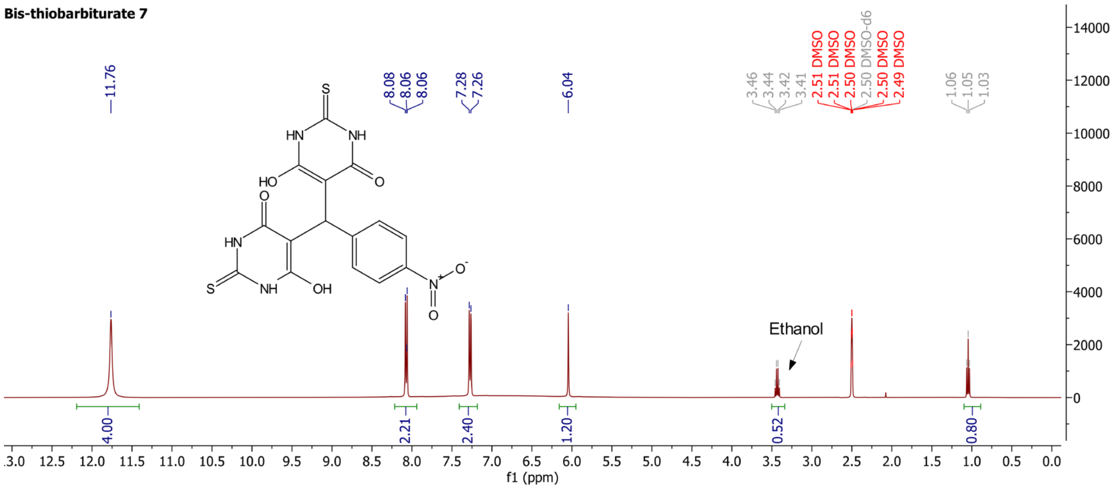

Figure S12 – ^{1}H NMR of bis-thiobarbiturate **7** in DMSO-*d*6.

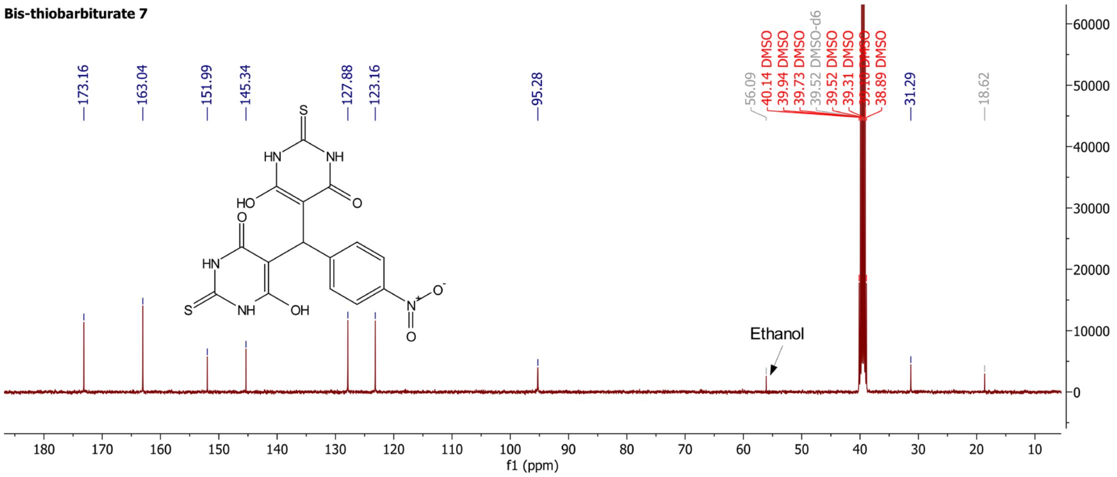

Figure S13 – ^{13}C NMR of bis-thiobarbiturate **7** in DMSO-*d*6.

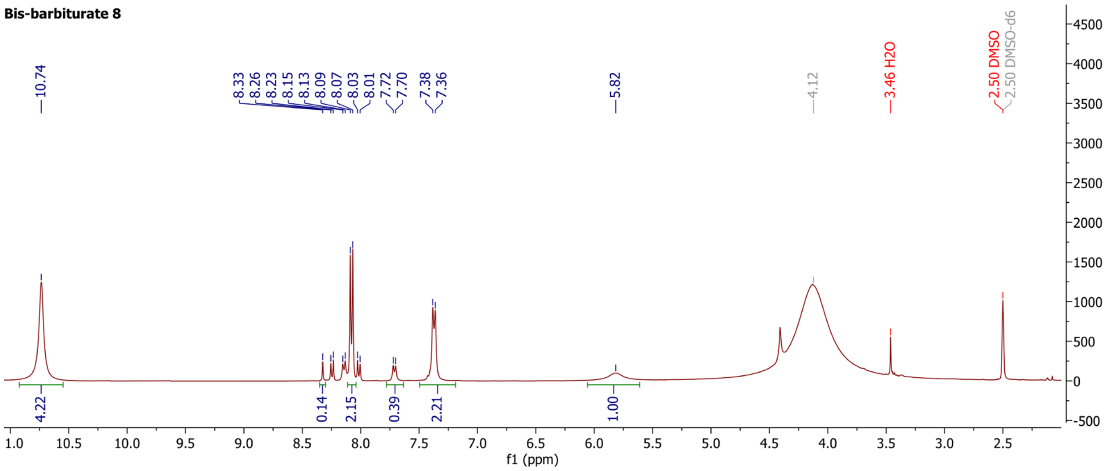

Figure S14 – ^{1}H NMR of bis-barbiturate **8** in DMSO-*d*6.

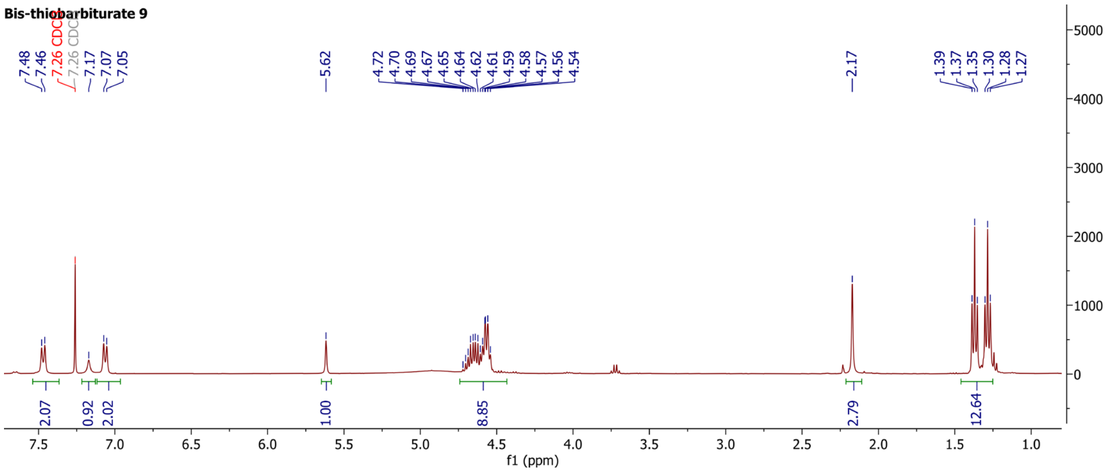

Figure S15 – ^{1}H NMR of bis-thiobarbiturate **9** in $CDCl_3$.

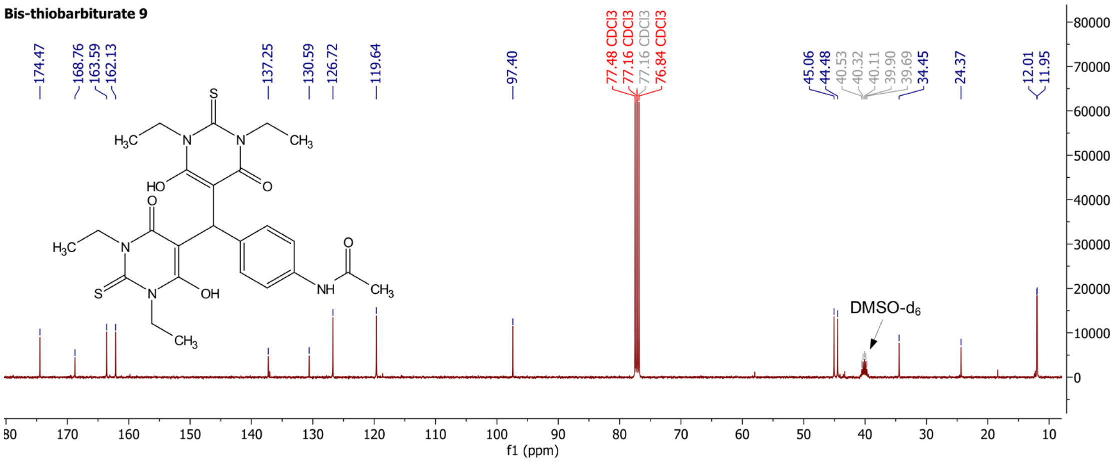

Figure S16 – ^{13}C NMR of bis-thiobarbiturate **9** in $CDCl_3$.

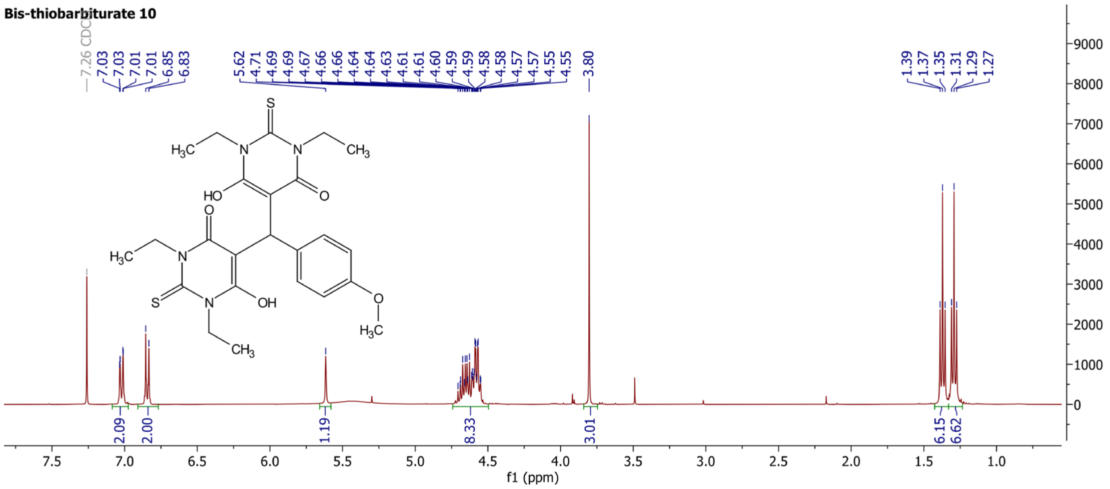

Figure S17 – ^{1}H NMR of bis-thiobarbiturate **10** in $CDCl_3$.

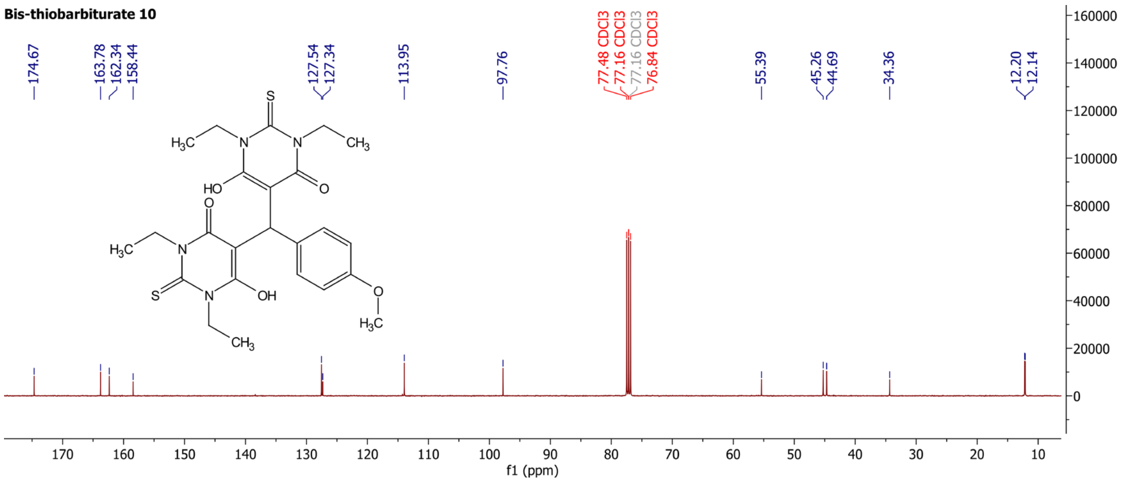

Figure S18 – ^{13}C NMR of bis-thiobarbiturate **10** in CDCl3.

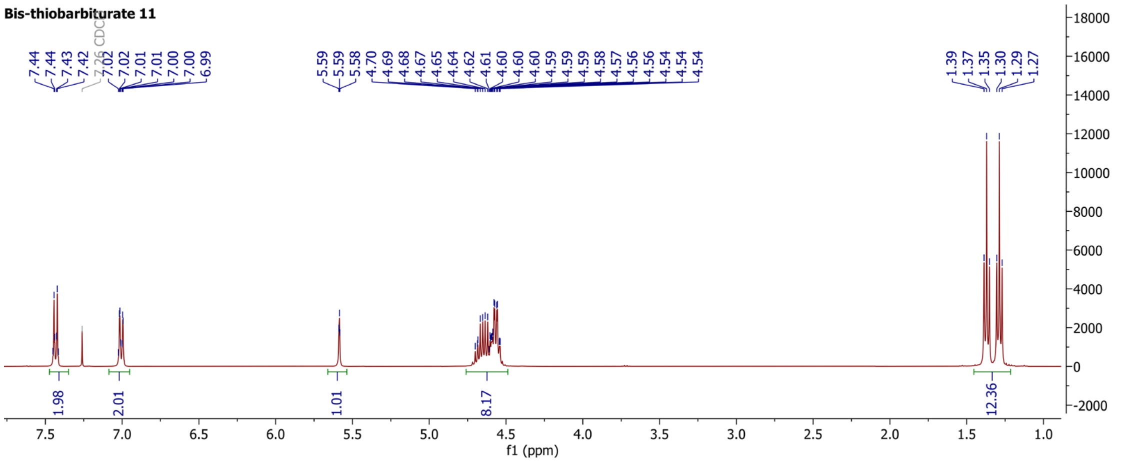

Figure S19 – ^{1}H NMR of bis-thiobarbiturate **11** in CDCl3.

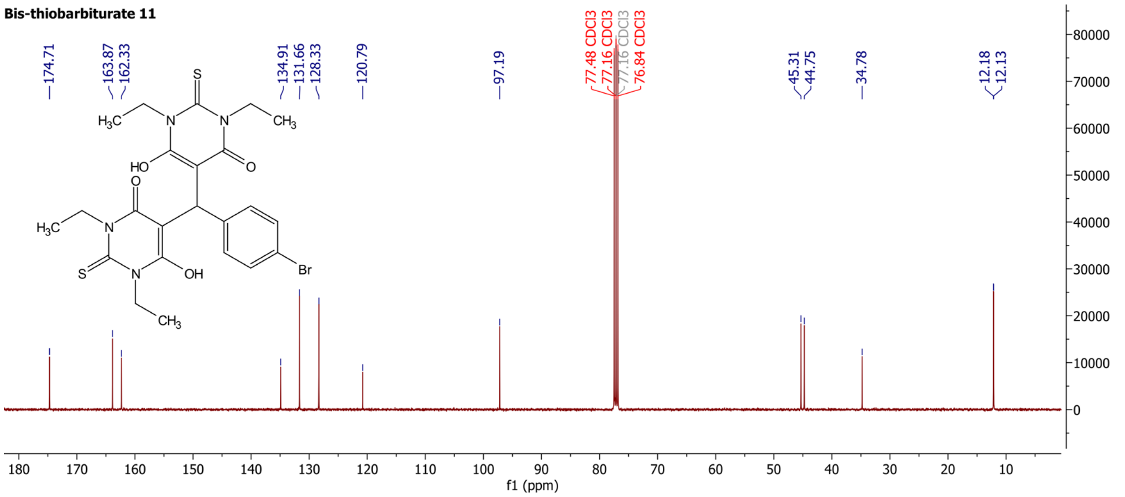

Figure S20 – ^{13}C NMR of bis-thiobarbiturate **11** in CDCl3.

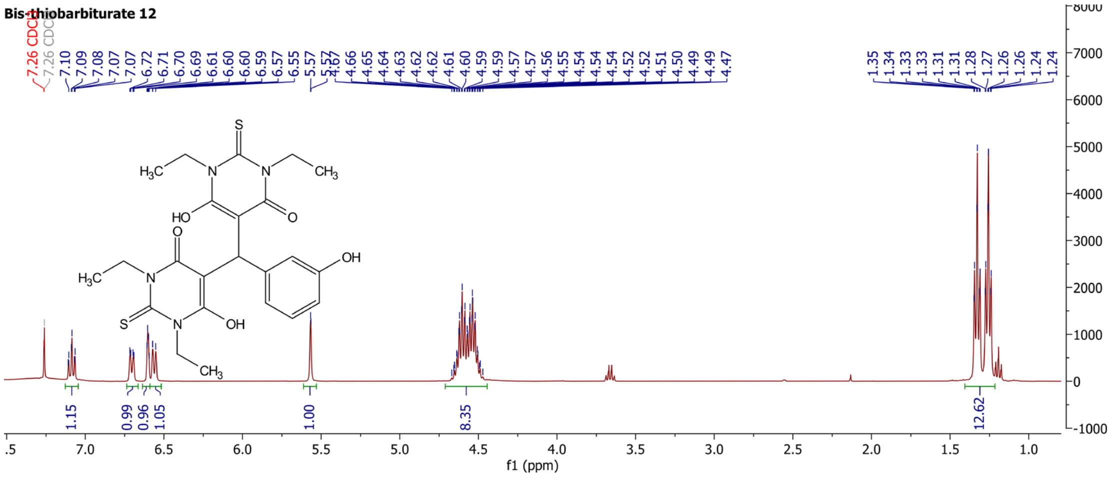

Figure S21 – ^{1}H NMR of bis-thiobarbiturate **12** in $CDCl_3$.

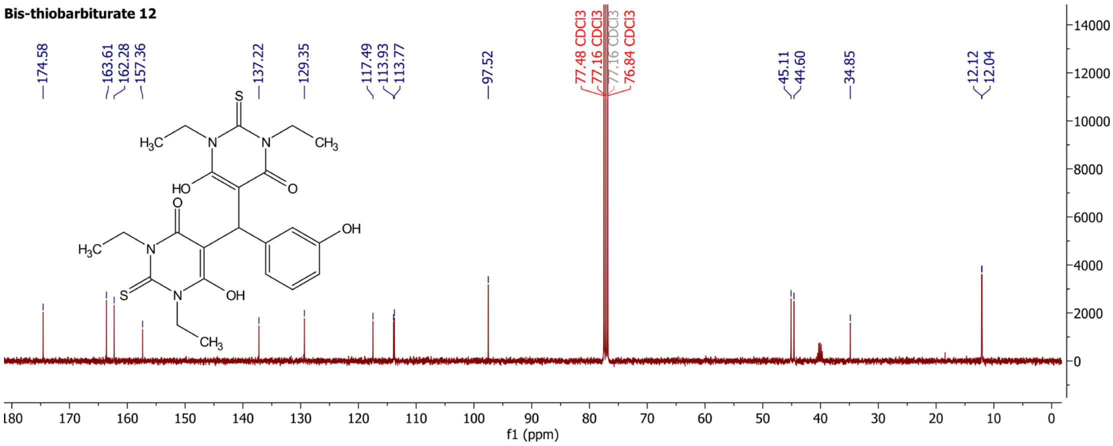

Figure S22 – ^{13}C NMR of bis-thiobarbiturate **12** in $CDCl_3$.

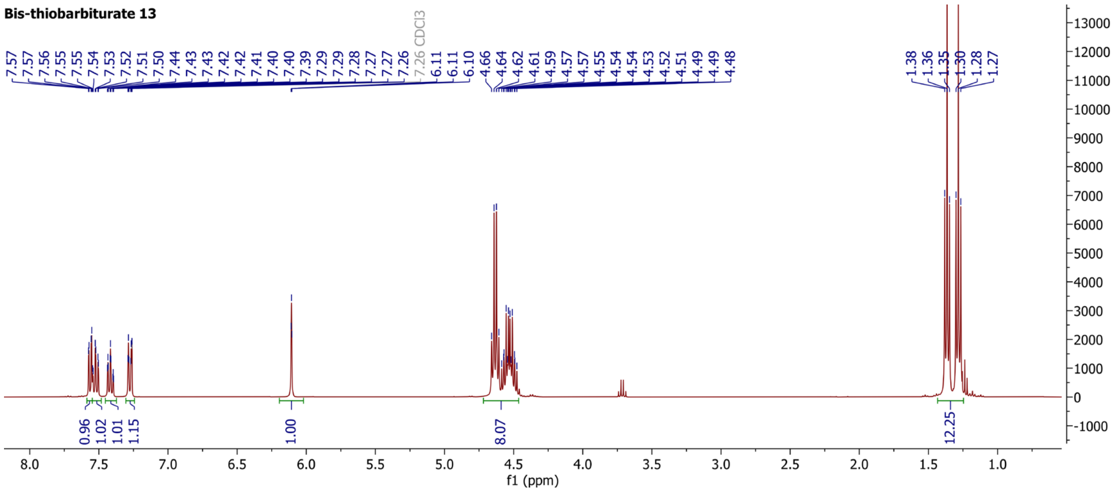

Figure S23 – ^{1}H NMR of bis-thiobarbiturate **13** in $CDCl_3$.

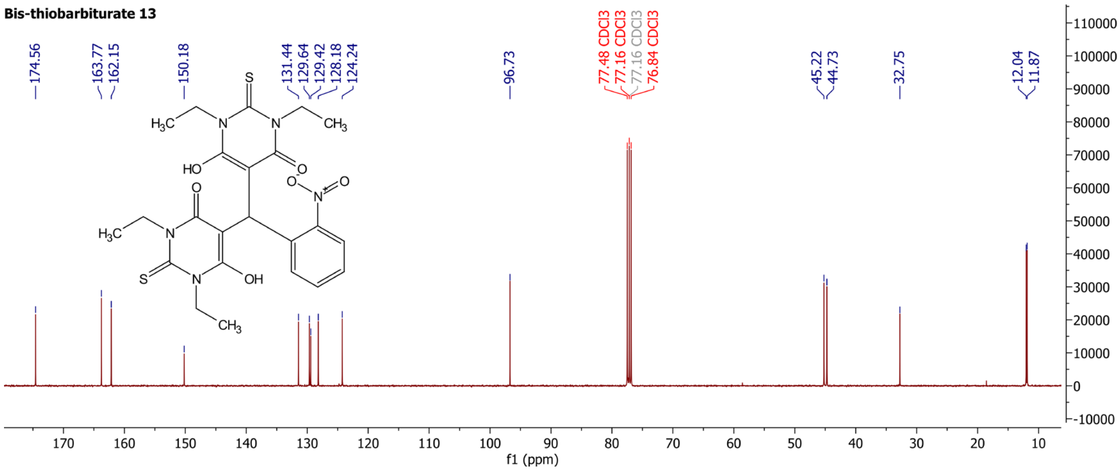

Figure S24 – ^{13}C NMR of bis-thiobarbiturate **13** in $CDCl_3$.

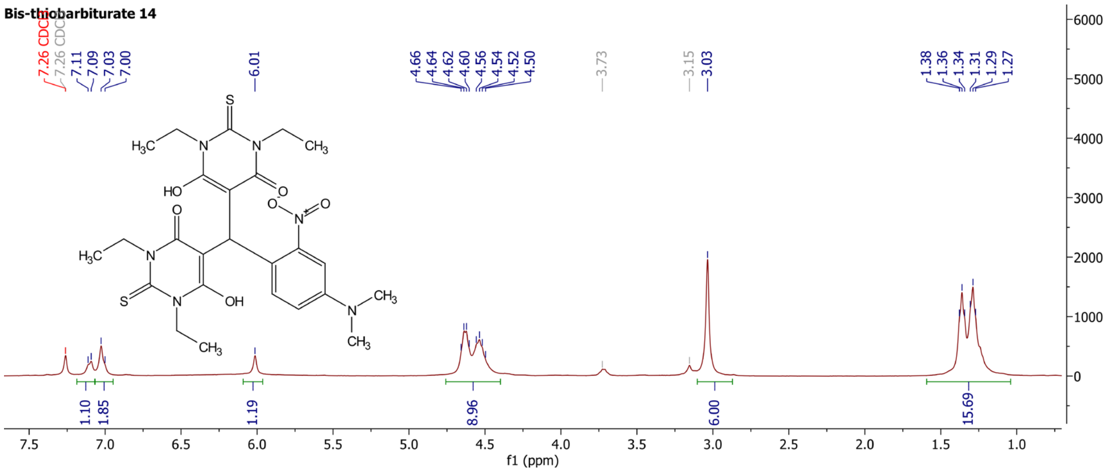

Figure S25 – ^{1}H NMR of bis-thiobarbiturate **14** in $CDCl_3$.

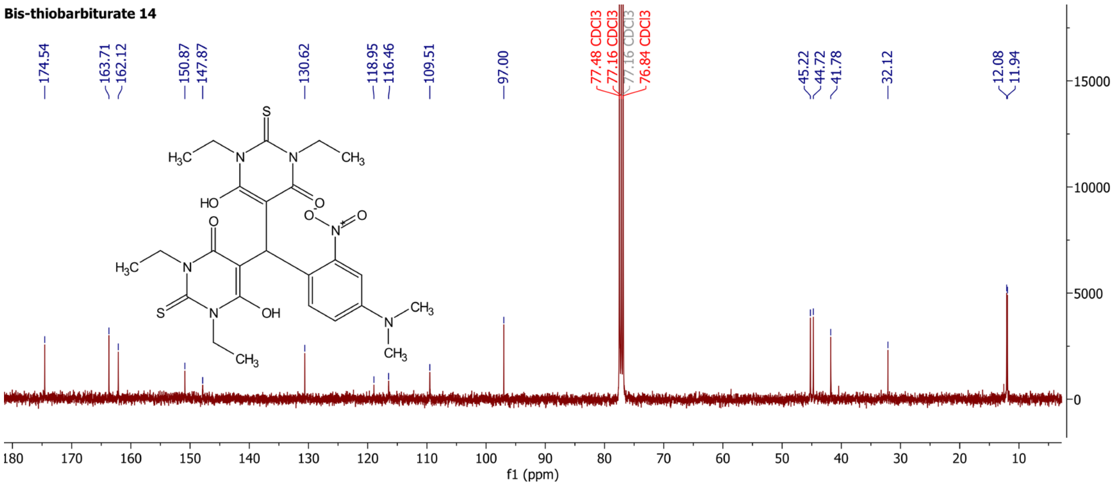

Figure S26 – ^{13}C NMR of bis-thiobarbiturate **14** in $CDCl_3$.

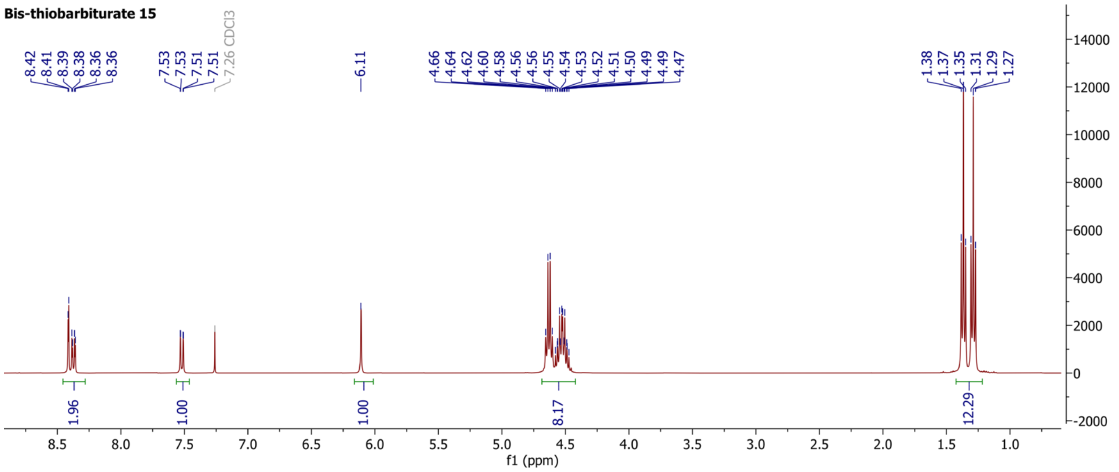

Figure S27 – ^{1}H NMR of bis-thiobarbiturate **15** in $CDCl_3$.

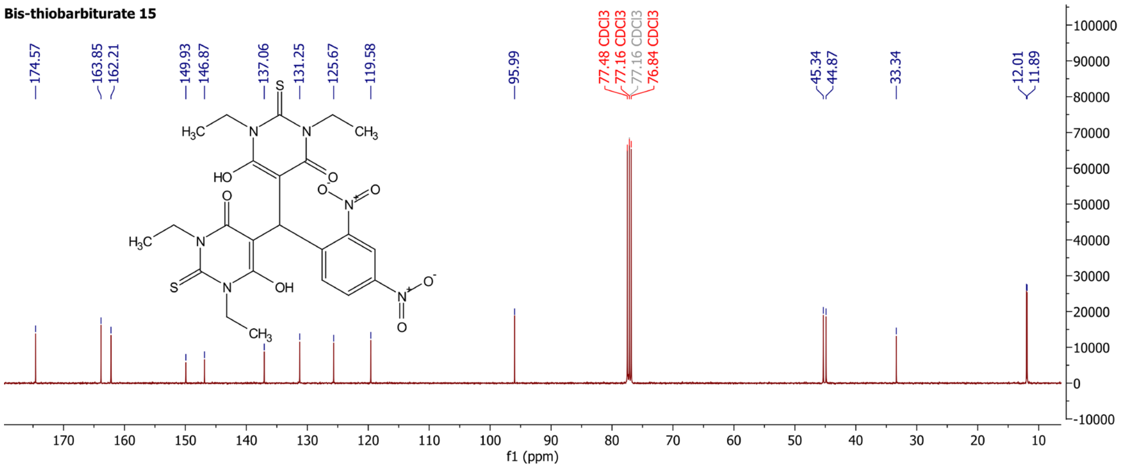

Figure S28 – ^{13}C NMR of bis-thiobarbiturate **15** in $CDCl_3$.

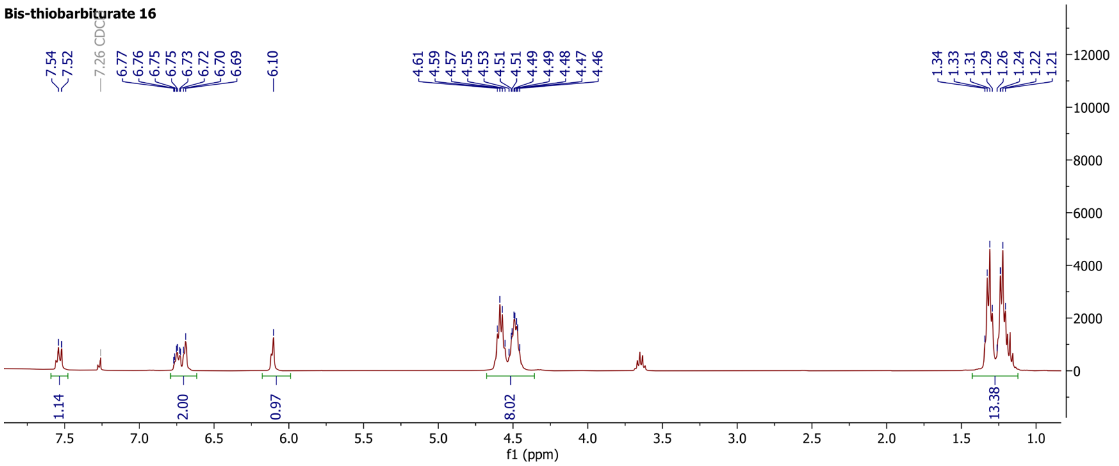

Figure S29 – ^{1}H NMR of bis-thiobarbiturate **16** in $CDCl_3$.

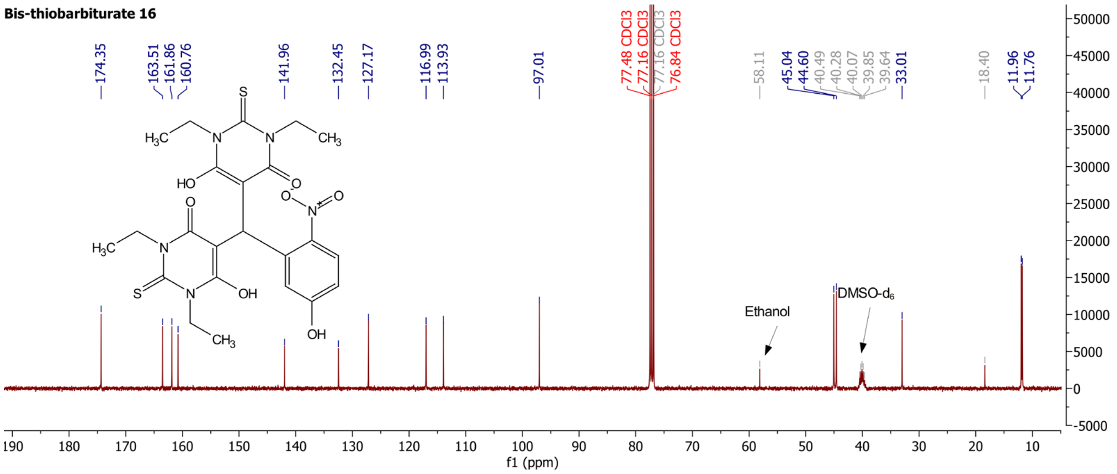

Figure S30 – ^{13}C NMR of bis-thiobarbiturate **16** in $CDCl_3$.

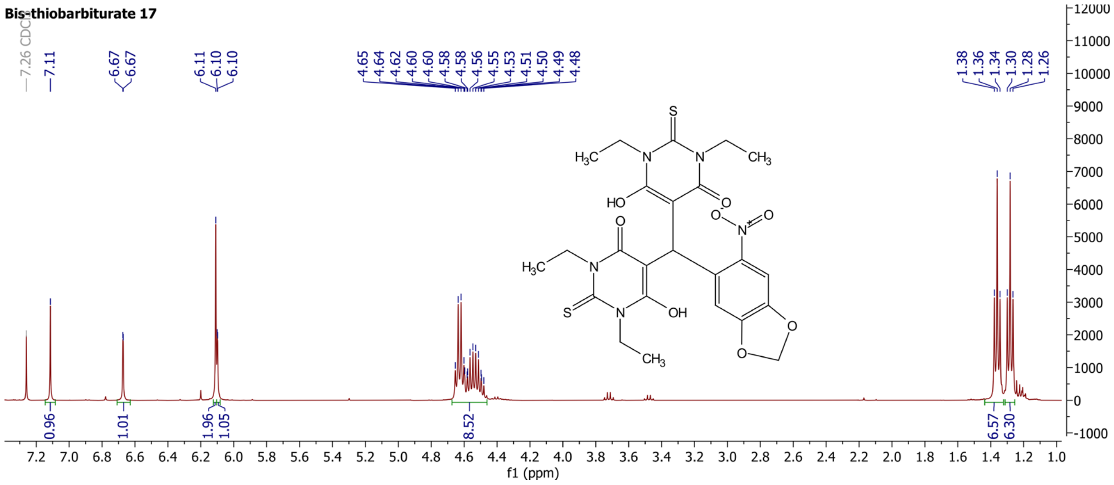

Figure S31 – ^{1}H NMR of bis-thiobarbiturate **17** in $CDCl_3$.

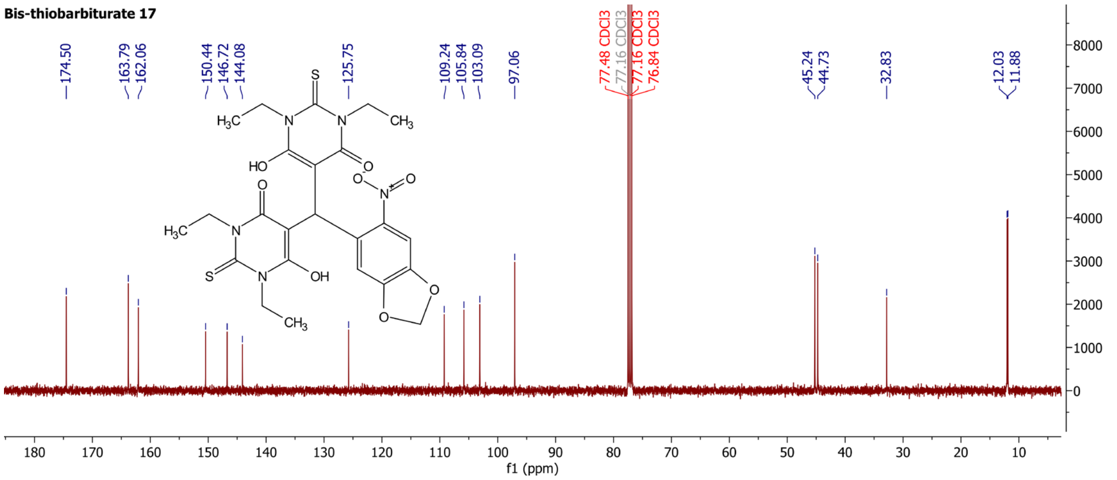

Figure S32 – ^{13}C NMR of bis-thiobarbiturate **17** in $CDCl_3$.

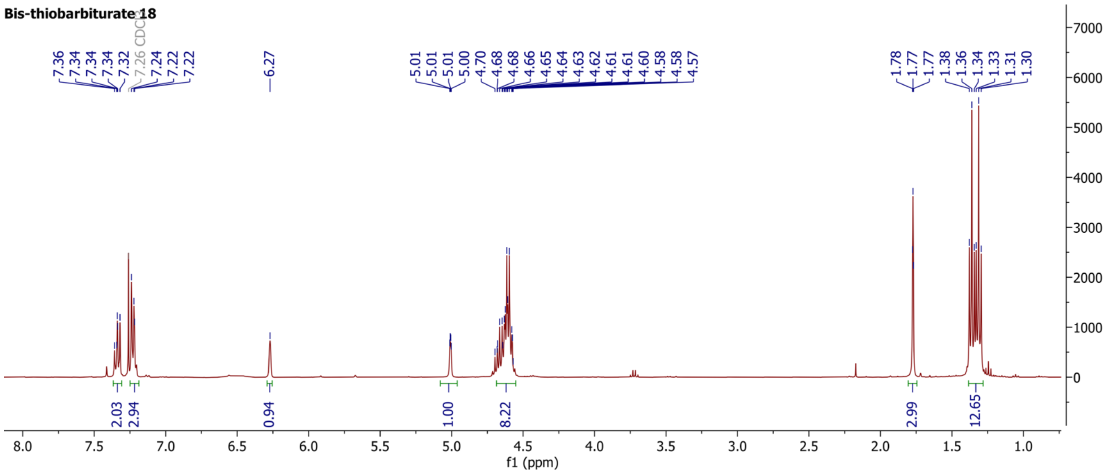

Figure S33 – ^{1}H NMR of bis-thiobarbiturate **18** in CDCl3.

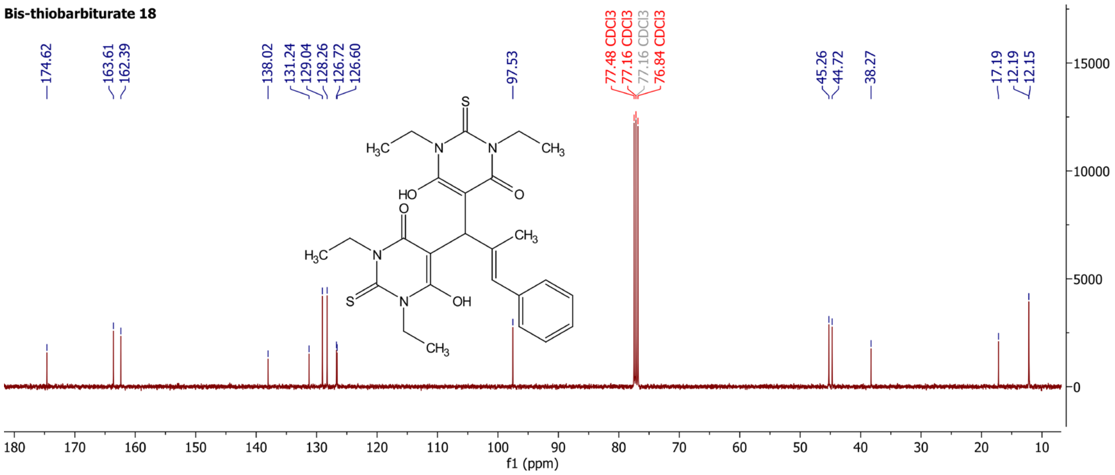

Figure S34 – ^{13}C NMR of bis-thiobarbiturate **18** in CDCl3.

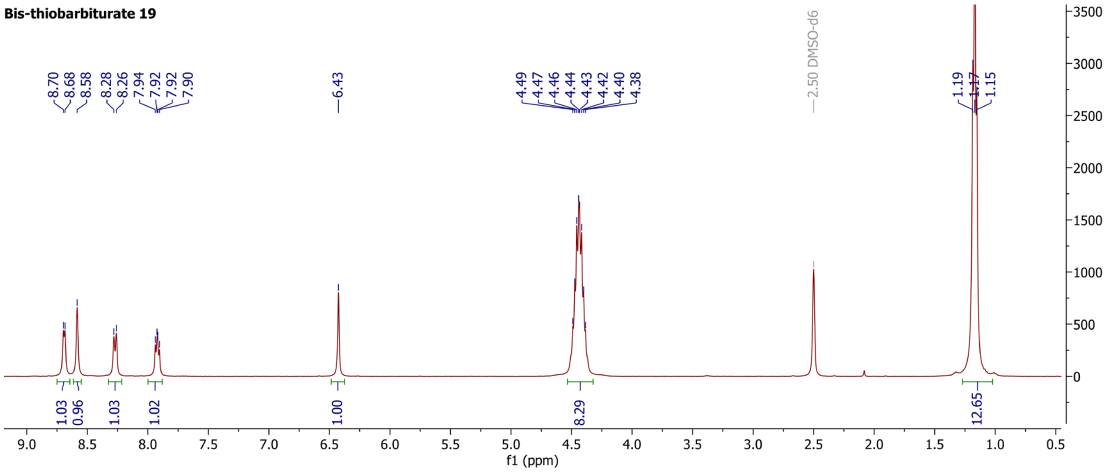

Figure S35 – ^{1}H NMR of bis-thiobarbiturate **19** in DMSO-d_6.

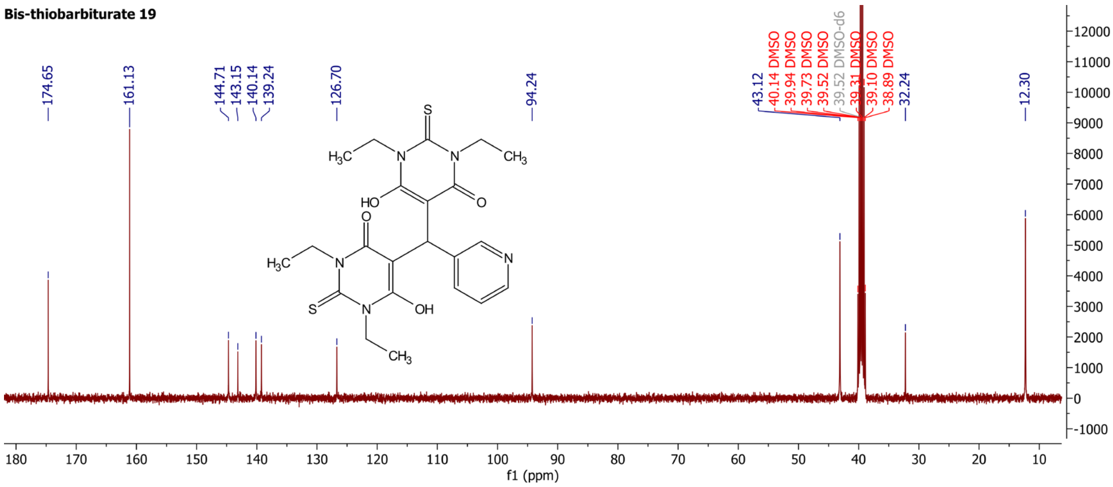

Figure S36 – ^{13}C NMR of bis-thiobarbiturate **19** in DMSO-d_6.
